# Blood Biomarker Profile Alterations in Newborn Canines: Effect of the Mother′s Weight

**DOI:** 10.3390/ani11082307

**Published:** 2021-08-05

**Authors:** Brenda Reyes-Sotelo, Daniel Mota-Rojas, Patricia Mora-Medina, Asahi Ogi, Chiara Mariti, Adriana Olmos-Hernández, Julio Martínez-Burnes, Ismael Hernández-Ávalos, Jose Sánchez-Millán, Angelo Gazzano

**Affiliations:** 1 Science Program “Maestria en Ciencias Agropecuarias”, Xochimilco Campus, Universidad Autónoma Metropolitana, Mexico City 04960, Mexico; breyess_20@yahoo.com.mx; 2 Neurophysiology, Behavior and Animal Welfare Assesment, DPAA, Xochimilco Campus, Universidad Autónoma Metropolitana, Mexico City 04960, Mexico; 3 Facultad de Estudios Superiores Cuautitlán, Universidad Nacional Autónoma de Mexico (UNAM), Cuautitlán Izcalli 54714, Mexico; mormed2001@yahoo.com.mx (P.M.-M.); mvziha@hotmail.com (I.H.-Á.); jlsan@unam.mx (J.S.-M.); 4 Department of Veterinary Sciences, University of Pisa, 56124 Pisa, Italy; asahi.ogi@vet.unipi.it (A.O.); chiara.mariti@unipi.it (C.M.); angelo.gazzano@unipi.it (A.G.); 5 Division of Biotechnology-Bioterio and Experimental Surgery, Instituto Nacional de Rehabilitación Luis Guillermo Ibarra Ibarra (INR-LGII), Mexico City 14389, Mexico; adrianaolmos05@gmail.com; 6 Animal Health Group, Facultad de Medicina Veterinaria y Zootecnia, Universidad Autónoma de Tamaulipas, Victoria City 87000, Mexico; jmburnes@docentes.uat.edu.mx

**Keywords:** animal perinatology, asphyxia, physiological blood profile, puppy welfare, stillbirth

## Abstract

**Simple Summary:**

Morphological variability in canines is associated with the mother’s size and weight, which likely affects the birth weight of the puppies and their metabolic status. Identifying physio-metabolic alterations in the blood from the umbilical vein to evaluate the concentration of gases, glucose, lactate, calcium, hematocrit levels, and blood pH of newborn puppies will make it possible to determine the risk of complications due to intrauterine asphyxia. The objective of this study is to evaluate the effect of the mother’s weight on the weight of liveborn and stillborn puppies during spontaneous births and the neonates’ blood physiological alterations during the first minute of life. The above allowed us to identify the physio-metabolic maladjustments that newborn puppies suffer from and to determine the risk of asphyxia according to the weight category of the mothers. Results suggest that if the weight of the bitch is >16.1 kg in eutocic births, there is a higher risk of intrapartum physiological alterations and death. The results of this study allowed us to identify that the weight of dams before birth determines the weight of the puppies at birth, though there is a wide range in birth weights due to the ample morphological variability characteristics of this species.

**Abstract:**

This study aims to determine the effect of the weight of bitches on liveborn and stillbirth puppies from eutocic births, and physiological blood alterations during the first minute postpartum. A total of 52 female dogs were evaluated and distributed in four categories: C1 (4.0–8.0 kg, n = 19), C2 (8.1–16.0 kg, n = 16), C3 (16.1–32.0 kg, n = 11), and C4 (32.1–35.8 kg, n = 6). The dams produced 225 liveborn puppies and 47 were classified as stillbirth type II. Blood samples were taken from the umbilical vein to evaluate the concentration of gases, glucose, lactate, calcium, hematocrit levels, and blood pH. The liveborn puppies in C2, C3, and C4 had more evident physiological alterations (hypercapnia, acidosis) than those in C1 (*p* < 0.05). These signs indicate a process of transitory asphyxiation. The stillborn pups in all four categories had higher weights than their liveborn littermates. C3 and C4 had the highest mean weights (419.86 and 433.79 g, respectively) and mortality rates (C3 = 20.58%, C4 = 24.58%). Results suggest that if the weight of the bitch is >16.1 kg in eutocic births, there is a higher risk of intrapartum physiological alterations and death. The results of this study allowed us to identify that the weight of dams before birth determines the weight of the puppies at birth.

## 1. Introduction

Mortality in dogs during the neonatal period has been estimated to reach 40% [1]. Deaths may occur in the uterus, during expulsion, immediately postpartum, or during the first weeks of life [2,3,4], but the highest number of stillbirths occurs during birth [5] and the first 7 days of life [6]. Approximately 60% of these deaths are associated with intrapartum asphyxiation [7] caused by dystocic deliveries [5,6,8]. Asphyxia during the birthing process also negatively impacts the newborns’ adaptation to extrauterine life [9] by limiting their viability and vitality [6,10,11,12,13]. A high neurologic morbidity increases the risk of neonatal mortality [14]. The birthing process is the most critical phase for newborns [15] because the transition from fetus to neonate involves physiological, biochemical, and anatomical changes accompanied by flows of hormones that trigger the respiratory function, vascular changes, and the activation of energy metabolism [16,17]; additionally, the maternal behavior is critical for the parturition to take place in favorable conditions for the newborn puppy [18,19,20,21]. Studies of dogs have reported that a certain level of transitory asphyxiation occurs during delivery. Though this is normal, it produces hypercapnia and transitory acidosis in puppies [22,23]. If these conditions persist, they will alter gas exchange [24], delay the onset of respiration, and generate metabolic acidosis in newborns [25]. The challenges of the birthing process, together with these risk factors can determine the proportion of the liveborn (LP) vs. stillbirth (SB) puppies and the viability of the former [26,27,28]. Morphological variability in canines is associated with the mother’s size and weight [23], for these likely affect the birth weight of the puppies [26,28,29,30] and their metabolic status. In both veterinary and human perinatology, analyzing blood gases and metabolites has emerged as an important tool for evaluating newborns [13,31], but reports on dogs are scarce. Studying physiological indicators provides crucial information and allows researchers to estimate variations in oxygenation levels, metabolic profiles, and the acid–base balance [32] that help determine the level of fetal hypoxia suffered during birth. Gasometry allows the monitoring of the respiratory function by measuring the concentration of certain gases (pO_2_, O_2_ saturation (SaO_2_), pCO_2_) and blood pH [11,12,33,34,35,36] and the evaluation of the acid-base balance—to estimate the newborns’ metabolic status [13,33,37,38]. Variations in metabolite levels, including lactate, play an important role in metabolic acidosis [39,40] associated with hypoxic events [1,41], high blood glucose levels [36], and a general compensatory metabolism marked by excess base and bicarbonate in the blood [25]. Identifying physio-metabolic alterations in the blood of newborn puppies will make it possible to determine the risk of complications due to intrauterine asphyxia. However, evidence on hypoxia in canines, its effects, and its relations to the mother’s weight as a risk factor is scant or has not been fully evaluated. Thus, the objective of this study was to evaluate the effect of the mother’s weight on the weight of the LP and SB puppies during spontaneous births and the neonates’ blood physio-metabolic alterations during the first minute of life. The above allowed us to identify the physiological maladjustments that newborn puppies suffer and determine the risk of asphyxia according to the weight category of the mothers.

## 2. Materials and Methods

### 2.1. Infrastructure

A network of 10 veterinary clinics in Mexico City was organized to recruit pregnant dogs. Prenatal control was performed from day 25 of pregnancy to 24 h postpartum.

### 2.2. Study Population

A total of 52 young multiparous bitches (2–4 births) were recruited. The inclusion criteria were: (i) clinically healthy dogs; (ii) valid vaccination/deworming record; (iii) fed a commercial formula; (iv) no history of reproductive problems; (v) radiographic and ultrasonographic evaluations to show they were apt for natural births. The exclusion criteria were: (i) primiparous females; (ii) bitches with a history of dystocia or pyometra; (iii) previous type I SB puppies; (iv) malformed fetuses; (v) use of birth inducers or accelerators; (vi) bitches with body condition 8 or 9 (obese) as per the WSAVA scale [42]; (vii) extremely aggressive behavior. Brachycephalic and large breeds were excluded due to their reported high incidence of dystocia [3]. The 52 pregnant females were classified in 4 categories according to their weight recorded before labor (day 60 ± 2), as follows: C1 (4.0–8.0 kg, n = 19); C2 (8.1–16.0 kg, n = 16); C3 (16.1–32.0 kg, n = 11); C4 (32.1–35.8 kg, n = 6). The body weight ranges respected the general guidelines for breed size established by the Federation Cynologique Internationale (FCI) [43].

### 2.3. Clinical History

The clinical history of the dogs was compiled, including age, weight, alimentation, preventive medicine status, and a description of the environment where they lived. All information was recorded in the Q.vet^®^ Ed. Professional 2016 database for veterinary clinics.

### 2.4. Diagnoses of Pregnancy

Diagnoses were confirmed between days 24 and 28 post-service for each dam. Fetal structures and cardiac activity were detected in the gestational sacs using a LOGIQ 400 MD ultrasound machine (General Electric, Yokohama, Japan) equipped with a 3.5 MHz convex transducer to establish probable due dates. Monitoring of fetal maturation and vitality was performed on days 40–50 of gestation. The fetal structure was defined completely to permit the early identification of pyometra cases, type I SB puppies, and malformations. X-rays of the dams’ abdomens were taken on day 45 of pregnancy once bone calcification of the fetuses was achieved to discard early stages of maternal–fetal dystocia and evidence of cephalopelvic disproportion, conditions that would make caesarean sections necessary [29]; thus, excluding the dam from the study. On day 60 of pregnancy, the females were checked by ultrasound to corroborate cardiac rhythms and fetal biparietal diameters. Monitoring of births was performed with a model S80Vet Sino-Hero^®^ vital sign monitor to evaluate the mothers’ physiological parameters. Clinical signs observed in the peripartum interval included anorexia, anxiety, and nesting behaviors.

### 2.5. Puppies

The number of LP and SB was recorded by category.

(a) Liveborn: A total of 225 LP puppies were recorded for the four categories: C1: 63; C2: 71; C3: 54; C4: 37. The neonates, who had a heartbeat and were breathing during the first minute of life, were considered liveborn puppies. The puppies that died once the birth was over, were considered dead during lactation.
(b) Stillbirth: The 47 SB, by the weight categories of their mothers, occurred as follows: C1: 9; C2: 12; C3: 14; C4: 12. The following cases were classified as dead antepartum (i.e., stillbirth Type I): fetuses that died after the birth process began but before the expulsion; those with hemorrhagic and edematous appearance; those with grayish-brown discoloration due to an initial state of mummification; more advanced cases, a clear state of dehydration and fur loss. Those fetuses were excluded. The fetuses classified as dead intrapartum (Type II SB) presented the same appearance as the rest of the litter, except for the absence of breathing and heartbeat.

#### 2.5.1. Blood Physio-Metabolic Profiles

##### Blood Sampling

A trained veterinarian took blood samples immediately after birth in less than 10 s. An assistant held the puppy in a supine position and exposed the abdominal region; the pup’s umbilical cord was carefully grasped to insert the needle (26G) of a tuberculin syringe impregnated with lithium heparin to avoid coagulation and alterations of blood values and immediately obtain 0.3 mL samples of venous blood. All samples were processed by a GEM Premier^®^ critical blood variable analyzer (Instrumentation Laboratory Diagnostics USA/Italy) to obtain values for the metabolite’s glucose (mg/dL) and lactate (mg/dL), blood gases pCO_2_ (mmHg) and pO_2_ (mmHg), the acid–base balance pH, HCO_3_^−^ (mmol/L), EB (mEq/L), Ca^2+^ (mmol/L), and hematocrit (Htc %). All profiles were tested for each LP and Type II SB pup.

#### 2.5.2. Birth Weight

The weight of each LP and Type II SB puppy was recorded using a digital scale (Salter Weight Tronix Ltd., West Bromwich, UK) after drawing the blood samples.

### 2.6. Statistical Analyses

Descriptive statistics were obtained for the variables tested following the Origin Version^®^ 9 statistical package procedure. Normality tests were performed for all dependent variables to determine differences among the 4 categories (the dam’s weight was the independent variable) regarding the parameters of the blood physio-metabolic profile: pH, pO_2_ (mmHg), pCO_2_ (mmHg), glucose (mg/dL), Ca^2+^ (mmol/L), lactate (mg/dL), hematocrit (Htc %), HCO_3_^−^ (mmol/L), and EB (mEq/L), as well as the weight of the LP pups (dependent variable). An analysis of variance (ANOVA) was performed with a contrast of means using a Tukey test (*p* < 0.05).

Values were considered as significant at *p* < 0.05.

*Metabolites*_*ijk*_ = *μ* + *T*_*i*_ + *CN*_*i*_ + *P*_*i*_ (*T*_*i*_*CN*_*i*_*P*_*i*_) + *e*_*ijk*_

where:

*Metabolites* = pH, pCO_2_, pO_2_, glucose, Ca^2+^, lactate, hematocrit, HCO_3_^−^, EB;
*µ* = general mean;
*T_i_* = fixed effect;
*CN_i_* = 1,2,3,4; for the case of SB Type II;
*P_i_* = birth weight;
*e* = error.

The same statistical model was used to analyze the SB Type II neonates.

### 2.7. Ethical Note

The studies were performed with privately owned dogs. Each owner gave her/his informed consent before data were gathered. During the study, all dogs were treated following the directives and guidelines of Mexico’s Official Norm NOM-062-ZOO-1999 on technical specifications for the production, care, and use of laboratory animals and those related to the field of applied etiological studies [44]. The experimental protocol (code CAMCA.32.18) was approved by the Committee of the Master’s Program in Agricultural Sciences of the Universidad Autónoma Metropolitana-Xochimilco, Mexico City.

## 3. Results

### 3.1. Weight

Significant statistical differences were observed (see Table 1, mean and standard error of the birth weights of neonates) between the weight of the LP puppies in categories one (189.85 ± 16.50 g; *p* = 0.01), two (266.84 ± 16.23 g; *p* = 0.01), and three (374.57 ± 48.18 g; *p* = 0.0001), with those in C1 having lower weights than those in C2 and C3 (76.99–184.72 g). There were no significant differences in the weight of the LP pups in C3 (374.57 ± 48.18 g) and C4 (381.02 ± 20.24) (*p* = 0.70).

Table 1 presents data on the weight of the Type II SB pups. The pups in C1 (219.11 ± 23.05 g; *p* = 0.01) weighed less than those in C2 (297.08 ± 17.62 g; *p* = 0.001), C3 (419.86 ± 4.57 g; *p* = 0.001), and C4 (433.75 ± 12.98 g; *p* = 0.001), but there were no significant differences between the weight of the SB pups in C3 (419.86 ± 4.57 g) and C4 (433.75 ± 12.98 g) (*p* = 0.40).

The lowest number of SB occurred in C1 with nine (12.5%), compared to C2, C3, and C4, which had 14.45–24.48%.

### 3.2. Blood Physiometabolic Profiles

The effect of the mother’s weight on the blood physio-metabolic profiles of LP and SB are shown in Table 2 and Table 3, respectively. In general, the weight category of the dam was related to metabolic changes in some of their puppies’ critical blood variables.

#### 3.2.1. Energy Metabolism

In terms of the level of blood lactate (see Table 2), there was a significant increase of 9.26–14.33% between the LP in C1 (4.80 ± 0.23 mg/dL, *p* = 0.001) and those in C2 (6.28 ± 0.17 mg/dL, *p* = 0.001), C3 (7.05 ± 0.27 mg/dL, *p* = 0.01), and C4 (7.27 ± 0.30 mg/dL, *p* = 0.01). The LP in C4 had the largest increase in blood lactate concentrations compared to C1 and C2 (*p* < 0.05), though there was no significant difference in lactate values between C2 and C3 (6.28 ± 0.17 mg/dL and 7.05 ± 0.27 mg/dL, respectively) (*p* = 0.07) (Table 2).

The Type II SB pups in C4 (13.08 ± 0.41 mg/dL) showed a larger increase in lactate than C1 (11.44 ± 0.52) (*p* = 0.02), but there were no significant differences between C2 and C3 (12.58 ± 0.31 and 12.5 ± 0.22, respectively) (*p* = 0.99) (Table 3).

In terms of blood glucose levels for the LP, no significant differences among the categories were found (*p* > 0.05) (Table 2). In contrast, the Type II SB pups in C1 (51.11 ± 3.18 mg/dL) had higher glucose levels than those in C3 (38.78 ± 3.97) (*p* = 0.04). There were no significant differences between C2 and C4 (42.58 ± 2.19 mg/dL and 41.91 ± 2.09 mg/dL, respectively) (*p* = 0.99) (Table 3).

#### 3.2.2. Calcium and Hematocrit

Regarding the metabolite Ca^2+^, a statistically significant decrease was found in the values of the LP in C1 (1.43 ± 0.01 mmol/L) compared to C2 (1.55 ± 0.01 mmol/L, *p* = 0.001), C3 (1.62 ± 0.01 mmol/L, *p* = 0.01), and C4 (1.59 ± 0.02 mmol/L, *p* = 0.01), but no significant differences in blood Ca^2+^ concentrations were seen among C2, C3, and C4 (*p* > 0.05) (Table 2). For the Type II SB, Ca^2+^ levels showed no significant differences among the categories (*p* > 0.05) (Table 3). 

Hematocrit values showed that the LP from the C2 (48.65 ± 0.41, 8.5%; *p* = 0.001), C3 (50.43 ± 0.39, 12.46%; *p* = 0.01), and C4 (49.82 ± 0.55, 11.1%; *p* = 0.01) dams all had higher percentages of hematocrit than those in C1 (44.84 ± 0.55) (*p* < 0.05). However, no significant differences were seen among the LP in C2, C3, and C4 (*p* > 0.05) (Table 3). Regarding the Type II SB, there were no significant differences among the categories for this value (*p* > 0.05) (Table 3).

#### 3.2.3. Acid–Base Balance

Observations showed that blood pH had a lower imbalance in the LP from the C1 dams (7.38 ± 0.01; *p* < 0.05), compared to C3 (7.29 ± 0.01; *p* = 0.01) and C4 (7.31 ± 0.02; *p* = 0.04). While pH clearly decreased, there were no significant differences in pH values among categories C2, C3, and C4 (*p* < 0.05) (Table 2). Similarly, for the Type II SB, there were no significant differences among the categories in terms of pH (*p* > 0.05) (Table 3). 

Regarding pO_2_, results showed that the LP from three groups—C2 (15.47 ± 0.36 mmHg, *p* = 0.01), C3 (14.48 ± 0.40 mmHg, *p* = 0.001), and C4 (15.18 ± 0.47 mmHg, *p* = 0.01)—had significant decreases compared to C1 (17.09 ± 0.44 mmHg) (*p* < 0.05), but there were no significant differences in the pO_2_ values among C2, C3, and C4 (*p* > 0.05) (Table 3). For blood pO_2_ values, we found that the Type II SB in C4 (5.75 ± 0.89 mmHg) had a significant decrease compared to C1 (9 ± 0.91 mmHg) (*p* = 0.02), but the pO_2_ values between C2 and C3 did not differ significantly (6.66 ± 0.63 mmHg, *p* = 0.15, and 6.21 ± 0.48 mmHg, *p* = 0.053, respectively) (Table 3). 

Regarding pCO_2_, a statistically significant increase was observed in the LP in C2 (54.42 ± 1.35 mmHg, *p* = 0.01), C3 (55.98 ± 1.63 mmHg, *p* = 0.001), and C4 (54.78 ± 1.87 mmHg, *p* = 0.01), compared to C1 (47.69 ± 1.01 mmHg). In contrast, no significant differences were found in the pCO_2_ values of C2, C3, and C4 (*p* > 0.05) (Table 2). Upon observing the blood pCO2 values in the Type II SB, a significant increase was found in groups C2 (93.08 ± 2.42 mmHg, *p* = 0.01), C3 (91.78 ± 2.28 mmHg, *p* = 0.01), and C4 (94.66 ± 1.9 mmHg, *p* = 0.01) compared to C1 (1.66 ± 2.08 mmHg), but no significant differences were found in the pCO_2_ values from groups C2, C3, and C4 (*p* > 0.05) (Table 3).

A significant decrease in HCO_3_^−^ levels was seen in the LP in C2 (20.44 ± 0.17 mmol/L, *p* = 0.001), C3 (19.96 ± 0.18 mmol/L, *p* = 0.01), and C4 (19.86 ± 0.21 mmol/L, *p* = 0.001) compared to C1 (21.94 ± 0.26 mmol/L) (*p* < 0.05), but upon comparing the metabolite values for C2, C3, and C4, no significant differences were found (*p* > 0.05) (Table 2). Regarding the SB, no differences were observed among the categories (*p* > 0.05) (Table 3). 

Observations showed that EB increased significantly in the LP in C4 (−8.82 ± 0.43 mEq/L) compared to the values for C1 (−4.77 ± 0.38 mEq/L, *p* = 0.01), C2 (−5.96 ± 0.36 mEq/L, *p* = 0.001), and C3 (−7.03 ± 0.45 mEq/L, *p* = 0.01). A similar result emerged when we compared the values for C1 and C3 (*p* = 0.001), though no significant differences were found between C2 and C3 (*p* = 0.11) (Table 2). Regarding the Type II SB, no significant differences were found among the categories (*p* > 0.05) (Table 3).

## 4. Discussion

### 4.1. Weight

It was proposed that the weight of puppies at birth can be influenced by diverse factors, as occurs in other mammals. These include the duration of pregnancy [27,45,46], restrictions on intrauterine growth [45,47,48], the mother’s nutritional status [27], breed [49], and the weight and size of the placenta [50]. However, the results of our study suggest that the mother’s weight before giving birth exerts an effect on the weight of the newborn puppies since this varied significantly among the four categories tested. A broad weight range was observed that might be attributable to this species’ extensive morphological variability characteristic [51]. The recorded weights of the 272 puppies born and classified according to the weight of the dam (based on FCI guidelines, [43]) showed a mean from 204.48 to 407.39 g (157–453 g), with a mean variation of 77.46 to 202.91 g. We believe that the weight of the neonates reflected the mother’s weight because our study did not consider breed as a variable. A study by Vassalo et al. [6] observed a similar effect, the mothers’ body weight influenced the weight of puppies born by eutocic births and cesarean section. While it is true that the dam’s nutritional status can affect the weight of the puppies—as occurs in humans [52]—this variable was not controlled in this study. 

One important finding involves the weight of the SB, as this was always higher than that of the LP in all four cases (C1–C4). These differences mean 29.16–52.77 g, but categories C3 and C4 had both the highest mean weights (419.86 and 433.79 g, respectively) and the highest mortality rates (C3 = 20.58%, C4 = 24.58%). On one side, and such as other species, including humans, swine, and bovines, low birth weight is considered an important risk factor for neonatal mortality [34,53,54]. Reports on dogs affirm that low birth weight is strongly related to mortality. In this regard, Groppetti et al. [55] and Mila et al. [56] pointed out that there is a twelve-fold higher mortality risk for the lightest puppies than those with normal weight. Though low birth weight has been deemed a disadvantageous condition for neonatal survival [3,55,57,58] and has been associated with a higher risk of fetal death, our study did not reveal signs of this because the heaviest puppies presented a higher risk of fetal death than those with the lowest weight (12.5 vs. 24.58% mortality). On the other side, previous results indicate that higher birth weights reduce postnatal mortality but increase the rate of intrapartum mortality due to the difficulties of birth caused by cephalopelvic disproportion and prolonged labor that can cause hypoxia or death [28,59,60]. The mortality rate in this study was 17.27%, counting only the pups that died intrapartum. While it is true that the cause of perinatal mortality in dogs is multifactorial, the mother’s weight must be considered a risk factor due to its impact on the weight of type II SB puppies, physiological alterations, and the acid–base imbalance present in puppies born in natural births.

### 4.2. Physiometabolic Profiles

Fetuses commonly suffer intermittent periods of light hypoxia due to uterine contractions and the mechanical pressure inherent to the birth process [61]. Vassalo et al. [6] affirmed that a state of fetal hypoxia during the perinatal period is common in newborn puppies. We measured the physiological and metabolic changes that LP experienced during eutocic births, including increases in blood levels of pCO_2_ and lactate of 17.38% and 51.45%, respectively, and decreases in blood pH of 1.22%, and levels of pO_2_ (15.27%) and bicarbonate HCO_3_^−^ (9.48%), with high EB (45.91%), all due to hypercapnia (an indicator of respiratory acidosis) as the main factor. The most evident alterations occurred in the LP groups with the heaviest dams (C2, C3, C4). These alterations in gases and blood metabolites indicated respiratory and metabolic acidosis (mixed acidosis) resulting from intermittent asphyxia in utero during natural birth. Compensatory alkalosis began as a response to the respiratory and metabolic acidosis in the LP in all groups (C1–C4) due to hypoxia. This explains why pH did not decrease drastically [62,63]. Hypoxia-induced stress increases circulating epinephrine that breaks down muscular glycogen; thus, increasing lactate concentrations [64,65,66]. The above slows metabolism and triggers delayed anaerobiosis, a mechanism known as a tolerance to fetal hypoxia [67].

Regarding the metabolite glucose, no significant differences were observed in the LP during the first minute of life in any of the four categories, since measurements were in a range of 94.92–103.91 mg/dL. Mila et al. [17] reported a mean plasma glucose concentration of 97 mg/dL between 10 min and 8 h postpartum. The blood glucose level of puppies in the first 24 h postpartum was established in a range of 88–133 mg/dL [68,69]. Adequate energy reserves are extremely important for neonatal survival and resistance to adverse climatic conditions [70]. However, during the first hours of life, a decrease in glucose concentrations may be seen in diverse species because the glucose supply is interrupted abruptly during birth. This decrease is associated with the rapid exhaustion of hepatic glycogen. Hypoglycemia increases blood glucagon, cortisol, and catecholamine levels, leading to gluconeogenesis, lipolysis, glycogenolysis, and the consumption of ketone bodies. Ingesting colostrum post-birth increases and maintains glucose levels. In humans, for example, values below 50 mg/dL have been observed, though these may increase to 81 mg/dL during daytime [71,72]. A similar situation has been seen in foals [73]. On another point, an increase in blood glucose in newborn piglets can be considered an accurate indicator of neonatal distress because it shows their incapacity to regulate, or compensate for, the physiological processes during birth [13,37,74]. Mota-Rojas et al. [75] mention that high glucose concentrations in piglets are a sign of a short episode of asphyxia compared to those that manage to maintain their energy reserves. Therefore, lower glucose levels are associated with a more extended period of asphyxia and higher a consumption of energy reserves. It is important to mention that a prolonged or intermittent asphyxia in utero during birth does not necessarily lead to intrapartum stillbirth. However, these conditions can weaken newborns and reduce their capacity to adapt to extrauterine life, as documented in piglet studies [36]. The events that occur during an acute process of asphyxiation—such as metabolic acidosis and hypoxia—impact the welfare of newborns and their postnatal development. 

Birth weight was reported as a risk factor for intrapartum hypoxia because newborns with a low birth weight are more likely to suffer oxygen restriction and the secondary effects of hypoxemia [76,77]. Present findings, however, indicate otherwise. The blood samples collected from the umbilical cords for the gas and metabolic analyses of the intrapartum SB indicated that the fetuses showed signs of severe metabolic acidosis moments before birth due to the low pH (range: 6.79–6.88), increased pCO_2_ levels up to double those registered in the LP (94.66 vs. 47.69 mmHg), lactate values as many as four times higher than in the LP (13.08 vs. 4.80 mg/dL), EB in a range of −14.26 to −15.33, and a decrease in pO_2_ (6.22%) and bicarbonate HCO_3_^−^ levels (6.97%). We also observed that the Type II SB showed a significant decrease in plasma glucose concentrations in every group (C1–C4), with a range of 38.78–51.11 mg/dL. This result coincides with the values < 40 mg/dL reported by Lawler [78], related to hypoglycemia, which indicate a depletion of the newborns’ energy reserves, as a by-product of a previous hypoxic process [7,78] accompanied by a delay in eliminating excess liquid from the lungs, a decrease in uterine blood circulation, deficient gas exchange, and an alteration of energy metabolism [16]. We, therefore, infer that prolonged uterine contractions without fetal expulsion, caused by the prolonged birth process characteristic of this species, trigger hypoxia by increasing anaerobic glycogenolysis and the development of metabolic acidosis, as has been seen in piglets [75,79,80], humans [81], foals [82], and domesticated animals, including buffaloes [83,84]. These are conditions that reduce vitality and increase mortality [85].

Fetal asphyxia, defined as a condition of hypoxemia with hypercapnia and acidosis, caused the death of the pups at birth in this study. Fetal asphyxia caused the death of 17.27% of the puppies in this study, but the highest percentages of intrapartum deaths were seen in C3 and C4, which together represented 55.31% of the mortality of the pups from natural births.

#### Acid–Base Balance

In LP, a pH below the reference values was observed (7.35–7.45) in C2, C3, and C4, but this parameter on its own does not permit measuring the accumulated exposure to hypoxia because it is expressed logarithmically [39]. It does, however, establish the existence of acidosis in newborns and reflects fetal hypoxic stress that occurred during birth. Hence, the relation among pH, bicarbonate, and pCO_2_ indicates a process of metabolic acidosis [86]. In addition, the concentration of excess base (EB) must be determined, as this is an indicator of a linear tendency that determines the accumulation of acidosis after being adjusted for variations in pCO_2_ [87]. 

The intense, constant uterine contractions necessary for fetal expulsion can compress the umbilical cord, drastically decreasing both placental and umbilical blood circulation [88]. The decrease in pO_2_ and the increase in pCO_2_ combined to lower the pH, but bicarbonate in the plasma mitigated this imbalance. As a result, the high concentration of bicarbonate generated a mixed acidosis that exacerbated the increase in ionized calcium and the decrease in protein-bonded calcium [89], producing hypocalcemia at birth. Andres et al. [25] indicate that delayed breathing and metabolic acidosis are related to mortality at birth. 

During pregnancy, fetuses depend on their mother for gas exchange and correct oxygenation through the placenta. This exchange is determined by the size of the fetuses, blood gas concentrations in the mother, and the latter’s capacity for transfer and transport. Thus, modifications to any of these parameters can generate a state of hypoxia and subsequent disruptions of the acid–base balance, such as metabolic acidosis [61]. It is believed that the fetus’ capacity to withstand birth stress and welfare depends on both its condition at birth and the birthing process itself (duration, number of contractions, fetus thermoregulation, physiological, and metabolic changes. In addition, the newborn has to make several adjustments to adapt to extrauterine life, such as maintaining normoglycemia, thermoregulation, etc.) [18,90,91,92,93,94,95]. 

## 5. Conclusions

The results of this study allowed us to identify that the weight of dams before birth determines the weight of the puppies at birth, though there is a wide range in birth weights due to the ample morphological variability characteristic of this species. We observed that the puppies born from bitches in categories three and four had the highest birth weights (397.21 and 407.39 g, respectively), with a difference greater than 100 g from the pups in C1 and C2. The LP puppies in category 1—the lightest ones (189.85 g)—had fewer physio-metabolic alterations during the first minute of life; while the litters that weighed 16.1–35.8 kg showed more physio-metabolic alterations, which impacted their adaption to extrauterine life and caused respiratory and metabolic acidosis and hypocalcemia, all of which contributed to the total mortality of 17% as a consequence of intrapartum hypoxia.

Furthermore, all the Type II SB were heavier (C1 > 29.26 g; C2 > 30.24 g; C3 > 45.29 g; C4 > 52.73 g) than the LP born in the same group. The above suggests that puppies with higher weights experience greater difficulty during expulsion through the birth channel, which increases the risk of suffering an acute process of intrauterine asphyxia that affects their chances of survival. The highest mortality rates for the heavier categories (C3 and C4) were 20.58% and 24.48%, respectively. Therefore, this study established the mother′s weight (16.1–35.8 kg) as a risk factor for fetal asphyxia in eutocic births and, consequently, a high percentage of intrapartum mortality. New studies will allow the integrated evaluation of other variables as risk factors related to in utero asphyxia in eutocic births and their influence on postpartum survival.

## Figures and Tables

**Table 1:** Mean and standard error of the birth weights of the liveborn (LP) puppies and the Type II stillbirth (SB) during the first minute of life, grouped according to the category of the mother.

Category	LP n°	Mean Weight (g) ± SEM	SB Type II n°	Mean Weight (g) ± SEM
**C1**	63	189.85 ± 2.07 *^a^*	9	219.11 ± 7.68 *^a^*
**C2**	71	266.84 ± 1.92 *^b^*	12	297.08 ± 5.08 *^b^*
**C3**	54	374.57 ± 6.55 *^c^*	14	419.86 ± 3.62 *^c^*
**C4**	37	381.02 ± 3.32 *^c^*	12	433.75 ± 3.74 *^c^*

*^a^*^,*b*,*c*^ letters indicate differences in the categories of the mother (weight). Variance analysis (*p* < 0.05); Tukey test for independent samples (*p* < 0.05). SEM—standard error of the mean. Weight of the mothers according to category: C1 (4.00–8.00 kg), C2 (8.10–16.00 kg), C3 (16.10–32.00 kg), C4 (32.10–35.8 kg).

**Table 2:** Mean and standard error of the blood physio-metabolic profile of the LP puppies grouped according to the category of the weight of the mother.

	Metabolites	C1 LP = 63 (Mean ± SEM)	C2 LP = 71 (Mean ± SEM)	C3 LP = 54 (Mean ± SEM)	C4 LP = 37 (Mean ± SEM)
Energy metabolism	Lactate (mg/dL)	4.80 ± 0.23 ^a^	6.28 ± 0.17 ^b^	7.05 ± 0.27 ^b,c^	7.27 ± 0.30 ^c^
	Glucose (mg/dL)	94.92 ± 1.76 ^a^	101.29 ± 2.11 ^a^	100.27 ± 3.05 ^a^	103.91 ± 3.69 ^a^
Calcium and hematocrit	Ca^2+^ (mmol/L)	1.43 ± 0.01 ^c^	1.55 ± 0.01 ^b^	1.62 ± 0.01 ^a^	1.59 ± 0.02 ^a,b^
	Hematocrit (%)	44.84 ± 0.55 ^c^	48.65 ± 0.41 ^b^	50.43 ± 0.39 ^a^	49.82 ± 0.55 ^a,b^
Acid-base balance	pH	7.38 ± 0.01 ^a^	7.33 ± 0.01 ^a,b^	7.29 ± 0.01 ^b^	7.31 ± 0.02 ^a,b^
	pO_2_ (mmHg)	17.09 ±0.44 ^a^	15.47 ± 0.36 ^b^	14.48 ± 0.40 ^b^	15.18 ± 0.47 ^b^
	pCO_2_ (mmHg)	47.69 ± 1.01 ^b^	54.42 ± 1.35 ^a^	55.98 ± 1.63 ^a^	54.78 ± 1.87 ^a^
	HCO_3_^−^ (mmol/L)	21.94 ± 0.26 ^a^	20.44 ± 0.17 ^b^	19.96 ± 0.18 ^b^	19.86 ± 0.21 ^b^
	EB (mEq/L)	−4.77 ± 0.38 ^a^	−5.96 ± 0.36 ^a^	−7.03 ± 0.45 ^a,b^	−8.82 ± 0.43 ^c^

^a,b,c^ Letters indicate differences in the category of the mother (weight). Variance analysis (*p* < 0.05); Tukey test for independent samples (*p* < 0.05). SEM—standard error of the mean. LP—number of LP in the samples. Blood samples taken in a maximum of 10 s post-birth. Weight of the mothers according to category: C1 (4.0–8.0 kg), C2 (8.10–16.0 kg), C3 (16.10–32.0 kg), C4 (32.10–35.8 kg).

**Table 3:** Mean and standard error of the blood physio-metabolic profile of the Type II SB grouped according to the category of weight of the mother.

	Metabolites	C1 Type II SB = 9 (Mean ± SEM)	C2 Type II SB = 12 (Mean ± SEM)	C3 Type II SB = 14 (Mean ± SEM)	C4 Type II SB = 12 (Mean ± SEM)
Energy metabolism	Lactate (mg/dL)	11.44 ± 0.52 ^b^	12.58 ± 0.31 ^a,b^	12.5 ± 0.22 ^a,b^	13.08 ± 0.41 ^a^
	Glucose (mg/dL)	51.11 ± 3.18 ^a^	42.58 ± 2.19 ^a,b^	38.78 ± 3.97 ^b^	41.91 ± 2.09 ^a,b^
Calcium and hematocrit	Ca^2+^ (mmol/L)	1.85 ± 0.02 ^a^	1.89 ± 0.01 ^a^	1.89 ± 0.02 ^a^	1.85 ± 0.02 ^a^
	Hematocrit (%)	59.48 ± 0.52 ^a^	58.97 ± 0.77 ^a^	58.33 ± 0.90 ^a^	58.01 ± 1.15 ^a^
Acid-base balance	pH	6.79 ± 0.06 ^a^	6.80 ± 0.04 ^a^	6.83 ± 0.03 ^a,b^	6.88 ± 0.02 ^a^
	pO_2_ (mmHg)	9 ± 0.91 ^a^	6.66 ± 0.63 ^a,b^	6.21 ± 0.48 ^a,b^	5.75 ± 0.89 ^b^
	pCO_2_ (mmHg)	81.66 ± 2.08 ^b^	93.08 ± 2.42 ^a^	91.78 ± 2.28 ^a^	94.66 ± 1.98 ^a^
	HCO_3_^−^ (mmol/L)	17.63 ± 0.25 ^a^	18.80 ± 0.57 ^a^	18.37 ± 0.44 ^a^	18.45 ± 0.46 ^a^
	EB (mEq/L)	−14.42 ± 0.72 ^a^	−15.33 ± 0.48 ^a^	−14.26 ± 0.39 ^a^	−14.38 ± 0.86 ^a^

^a,b^ Letters indicate differences in the category of the mother (weight). Variance analysis (*p* < 0.05); Tukey test for independent samples (*p* < 0.05). SEM—standard error of the mean. LP—number of LP in the samples. SB—Stillbirth. Blood samples taken in a maximum of 10 s post-birth. Weight of the mothers according to category: C1 (4.0–8.0 kg), C2 (8.10–16.0 kg), C3 (16.10–32.0 kg), C4 (32.10–35.8 kg).

## Abbreviations/Nomenclature

pO_2_	partial oxygen saturation
O_2_	oxygen
EB	excess base
HCO_3_^−^	bicarbonate
Ca^2+^	calcio
Htc	hematocrit
